# Drug-mediated disruption of the aging gut microbiota and mucosal immune system

**DOI:** 10.3389/fragi.2025.1603847

**Published:** 2025-10-13

**Authors:** Lia Totleben, Joel Thomas, Daniel Austin

**Affiliations:** Lake Erie College of Osteopathic Medicine, School of Pharmacy, Erie, PA, United States

**Keywords:** aging, gut microbiota, mucosal immunity, antibacterials, PPIs, metformin, anti-inflammatory agents, corticosteroids

## Abstract

The human gut microbiota is comprised predominantly of bacteria, and also includes archaea, fungi, and viruses. The gastrointestinal epithelium, mucosal barrier, and mucosal immune system balance protection against infection at mucosal entry points with symbiosis and tolerance to non-harmful organisms and antigens. Aging is associated with notable changes in both gut microbiota and mucosal immunity, including reduced microbial diversity, increased proportion of pathobionts relative to commensals, immunosenescence, and chronic inflammation. These changes may disrupt gastrointestinal function and homeostasis and increase susceptibility to infection and inflammatory conditions. Multiple drug classes are also associated with disruption of the gut microbiota and mucosal immunity, including antibacterials, proton pump inhibitors (PPIs), metformin, and steroidal and non-steroidal anti-inflammatory agents. This review describes the mechanisms by which these drugs affect the gut microbiota and mucosal immunity to provide perspective of the concurrent effects of drugs and age-related changes.

## Introduction

### Overview of gut microbiota

The human gut microbiota functions as an intricate and diverse ecosystem composed of bacteria, archaea, fungi, and viruses, which collectively impact host digestion, metabolism, and immunity. The gut microbiota along with mucosal immunity provide colonization resistance to protect the host against pathogens at the mucosal surfaces. This relationship between the gut microbiota and host is critical for maintaining a commensal or mutualistic relationship, as well as host nutritional and immune homeostasis (Thursby and Juge, 2017). While there is significant variability, bacteria of the human gut microbiota are predominantly of the phyla Firmicutes, Bacteroidetes, Actinobacteria, Pseudomonadota (Proteobacteria), and sometimes Verrucomicrobia, and the population stays relatively constant over time for a given individual. The population can be disrupted or changed based on age, diet, antimicrobial exposure, and the immune response of the host (Hou et al., 2022).

### Firmicutes

Gut Firmicutes, primarily *Lactobacilli*, *Clostridia*, and *Enterococci* in, are Gram-positive bacteria that are essential for the fermentation of carbohydrates into short-chain fatty acids (SCFAs). These SCFAs serve a primary role in preserving and maintaining intestinal integrity along with regulating mucosal immunity. Numerous Firmicutes are probiotic organisms, often found in dietary supplements. The relative abundance of Firmicutes may be greater in obese individuals (Magne et al., 2020). They are susceptible to alterations by beta-lactams, glycopeptides, and fluoroquinolones, which can lead to dysbiosis and opportunistic infections (Induri et al., 2022).

### Bacteroidetes

Bacteroidetes, primarily *Bacteroides* and *Prevotella*, are significant in the fermentation of complex polysaccharides and the synthesis of SCFAs. Though essential for gut homeostasis, certain species can become pathogenic in dysbiotic conditions. Bacteroidetes are primarily targeted by broad-spectrum antibiotics such as carbapenems and fluoroquinolones, affecting the microbial balance (Weersma et al., 2020).

### Actinobacteria

Actinobacteria, such as *Bifidobacterium*, are important for the digestion of dietary fibers and immune response regulation. This bacterium is commonly found in probiotics and, in moderation, is known to promote intestinal health (Hidalgo-Cantabrana et al., 2017). They are susceptible to macrolides, penicillins, vancomycin, and other agents that cover gram positive bacteria (Esaiassen et al., 2017).

### Verrucomicrobia

Verrucomicrobia, primarily *Akkermansia muciniphila*, is one of few gut bacteria able to utilize mucin as a primary energy source. Mucin, a major component of the intestinal mucus layer, is known to contribute to the mucosal barrier integrity and metabolic health of the host. However, degradation by *A. muciniphila* is beneficial as it promotes mucus turnover and the release of short-chain fatty acids (Yan et al., 2021). This bacterium is sensitive to broad-spectrum antibiotics and is being studied for use in treatment of metabolic and inflammatory diseases (Zheng et al., 2023).

### Pseudomonadota (formerly Proteobacteria)

Bacteria of Pseudomonadota, such as *Escherichia* and *Helicobacter*, include beneficial and potentially pathogenic species, which may be associated with disease and inflammatory conditions. Pseudomonadota overgrowth, often associated with frequent antibiotic exposure or an immunosuppressive state, enhances gut inflammation (Esaiassen et al., 2017). Selected genera, role in gut health, and antibacterial therapy considerations for these phyla are contained in Table 1.

**TABLE 1:** Gut microbiota characteristics and functions Table 1. Major bacterial phyla of the gut microbiota and their primary roles in health and disease.

Phylum	Genera	Role in gut health	Dietary influence	Associated gut pathogenicity	Colonization resistance (mechanisms)	Antibacterial coverage	Probiotics use
Firmicutes	*Lactobacilli* *Clostridia Faecalibacteria* *Streptococci* *Peptostreptococci* *Eubacteria* *Bacillus*	SCFA production, carbohydrate fermentation, immune tolerance	Fiber promotes growth, high-fat diet increases abundance	Obesity Diabetes Inflammatory bowel disease (IBD) Mental health	Strong (SCFA production, biofilm, immune modulation, nutrient competition)	Generally susceptible to agents that cover Gram positive bacteria	*Lactobacillus* *Bacillus, Streptococcus, Enterococcus spp.*
Bacteroidetes	*Bacteroides Prevotella* *Porphyromonas*	Carbohydrate metabolism, SCFA production, regulation of mucin secretion	Fiber-rich diet enhances presence, high-fat diet decreases abundance	IBD Inflammatory bowel syndrome (IBS) Appendicitis Intra-abdominal abscess	Strong (SCFA production, immune modulation, bacteriocins, bile acid modification)	Generally susceptible to agents that cover anaerobic bacteria, high potential for acquired resistance	Uncommon
Actinobacteria	*Bifidobacteria* *Actinomyces*	Immune modulation, SCFA production, Gut permeability modulation	Fiber and prebiotics promote growth	Uncommon	Moderate (SCFA production, immune modulation, nutrient competition)	Generally susceptible to agents that cover Gram positive bacteria	*Bifidobacterium, Streptomyces spp.*
Pseudomonadota	*Escherichia Helicobacter Salmonella* *Citrobacter* *Proteus* *Enterobacter* *Klebsiella* *Vibrio* *Moganella* *Serratia* *Yersinia*	Diverse, may be beneficial or pathogenic, inflammation	Diet-dependent, high-fat diet may increase abundance	Nosocomial Enterocolitis Shanghai fever Necrotizing disease	N/A (Increased abundance associated with pathogenicity)	Antibacterial resistance is common	Uncommon
Verrucomicrobia	*Akkermansia*	Mucosal integrity, metabolic regulation	Fiber-rich diet supports growth	Uncommon	Moderate	May be susceptible to agents that cover Gram negative bacteria	Uncommon

The five predominant phyla—Firmicutes, Bacteroidetes, Actinobacteria, Proteobacteria, and Verrucomicrobia—have distinct metabolic and immune functions. Firmicutes are the main short-chain fatty acid (SCFA) producers and maintain barrier integrity (Induri et al., 2022). Bacteroidetes play a critical role in carbohydrate metabolism and SCFA production (Weersma et al., 2020). Actinobacteria, particularly Bifidobacterium, ferment carbohydrates and modulate host immunity (Clarke et al., 2019). Proteobacteria include opportunistic pathobionts such as *E. coli* that expand during dysbiosis and inflammation (Clarke et al., 2019). Verrucomicrobia, mainly Akkermansia muciniphila, support mucosal integrity and metabolic regulation (Cheng et al., 2024).

### Mucosal immunity

The mucosal immune system serves as the primary defense against intestinal pathogens while also maintaining tolerance to commensal or mutual microbes and dietary antigens. Many cells, substances, and processes are associated with maintaining this balance. Gut-associated lymphoid tissue (GALT), secretory immunoglobulin A (sIgA), epithelial cells, dendritic cells, toll-like receptors (TLRs), interleukins (ILs), inflammasomes, macrophages, and other chemical messengers collectively form an immunological barrier to pathogens while regulating the response of the adaptive immune system to microbiota (Tian et al., 2024). For example, sIgA may bind to, neutralize pathogens, and prevent microbial adhesion to epithelial surfaces, or present components of commensal bacteria to tolerogenic dendritic cells to reduce response. The diversity of organisms and need to determine their pathogenicity therefore presents a unique challenge with potential for dysbiosis either by unregulated bacterial growth and pathogenesis or excessive response with inflammation and damage to microbiota.

### Drugs and the aging gut microbiota

Immunosenescence and chronic inflammation (“inflammaging”) during aging contribute to microbial dysbiosis, reduced SCFA production, and weakened epithelial defenses, increasing susceptibility to infections (Zheng et al., 2022). Additionally, pharmaceutical interventions, including proton pump inhibitors (PPIs), metformin, nonsteroidal anti-inflammatory drugs (NSAIDs), corticosteroids, and antibiotics, further influence the gut microbiota and mucosal immune landscape. Risk of colorectal cancer (CRC), dysregulated growth and proliferation of cells of the inner lining of the colon or rectum, is higher in the aging population, although the incidence has increased in younger adults since the 1990s (Zaki et al., 2022). Microbial dysbiosis is a known risk factor for CRC, and the microbiota of CRC patients are distinct by overall composition and decreased diversity. Known bacterial associations include increased relative abundance of *F. nucleatum*, *E. faecalis*, *E. coli*, *P. anaerobius* in CRC patients, and decreased beneficial bacteria such as butyrate and lactate producers (Y. Cheng, et al., 2020). These associations occur at the population level, there is not one specific bacterial species or phylum is specifically associated with oncogenesis (the listed species are of members of Fusobacteriota, Firmicutes, Pseudomonadota, and Bacillota respectively), and further study is necessary to better understand the role of dysbiosis. Understanding the interactions between gut microbiota, mucosal immunity, aging, and medication use is crucial for developing targeted interventions that mitigate dysbiosis-related disorders. This review addresses drug-induced alterations in the gut microbiota impact mucosal immunity and aging-related changes, providing insights into strategies to preserve microbial and immune homeostasis.

## The aging gut microbiota and mucosal immunity

One of the most notable alterations in the aging gut microbiota is the reduction in microbial diversity. The decrease of beneficial bacterial species leads to a general decline in overall colonization resistance, making the gut more susceptible to inflammation and dysbiosis. Reduced production of short-fatty acid chains (SCFAs), which are essential for the epithelial integrity and mucosal immune response of the gut barrier, is a key change implicated in decreased colonization resistance (Zheng et al., 2022). Among the SCFA-producing gut bacteria, reduced abundance of *Bifidobacterium* (Actinobacteria) and *Clostridium* (Firmicutes) are particularly associated with age-related decline. The reduction of these microbes leads to compromised mucosal barrier function and increased gut permeability, which can promote systemic inflammation and age-related metabolic disorders like metabolic syndrome and type 2 diabetes. SCFA’s role in increasing colonocytes and immune response modulation is well known, while their reduction is linked to chronic low-grade inflammation, a hallmark factor of aging (Cheng et al., 2024). *Akkermansia muciniphila* (Verrucomicrobia), a key bacterium associated with gut barrier integrity and metabolic regulation, tends to decrease with age (Zeng et al., 2023). The loss of this bacterium is associated with an increase in gut permeability and enhanced inflammatory responses (Clark et al., 2022). *Lactobacillus (*Firmicutes), which is responsible for microbial homeostasis and the production of antimicrobials, also decreases with age, adding to gut dysfunction (Cheng et al., 2024).

**FIGURE 1:**
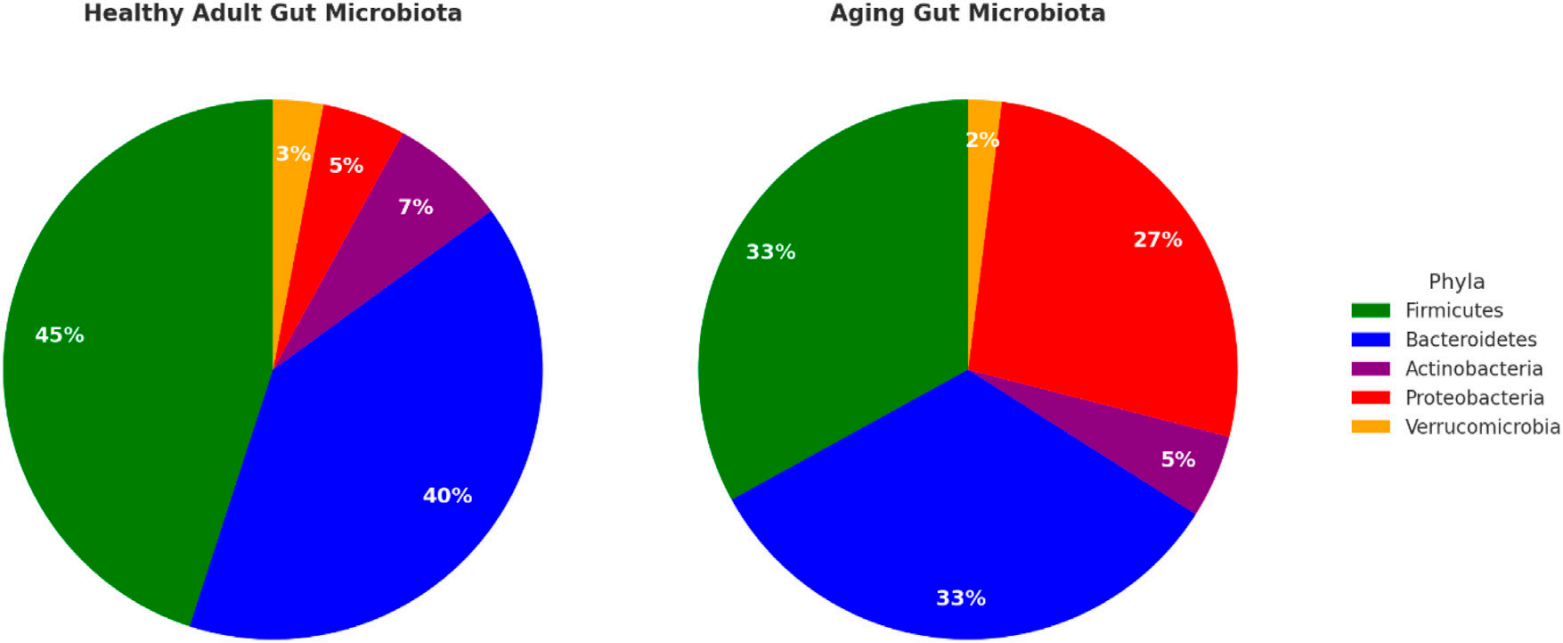
Gut microbiota composition in health vs aging comparison of the relative abundance of major gut bacterial phyla in adults versus the aging population. The relative abundance of firmicutes and bacteroidetes is grater, along with overall greater diversity at the genera and species level, in the adult microbiota. Data adapted from Induri et al. (2022); Weersma et al. (2020), Clarke et al. (2019), and Cheng et al. (2024).

The aging gut is also associated with an increase in pathobionts; microbes that have the potential to induce inflammation and disease in specific circumstances (Zhang et al., 2015). One such bacterium that shows an increase in the aging gut is *Escherichia coli* (Proteobacteria). An overgrowth of this microbe is often associated with antibiotic use, reduced immune surveillance, and shifts in gut pH, all of which are common in aging populations (Clark and Walker, 2018). These conditions lead to enhanced gut inflammation, increased intestinal permeability, and a greater risk of infections. These changes are depicted in Figure 1.

In conjunction with changes in microbial composition, immunosenescence, a decline in the efficiency of the immune system, is also associated with aging. For example, aging is associated with reduction in secretory immunoglobulin A (sIgA), which is involved with neutralizing pathogens, preventing their adhesion to epithelial cells, and maintaining microbial hemostasis. Reduction of this immunoglobulin can cause a weakening of the mucosal defense system and an increase in the risk of infections and dysbiosis (Zheng et al., 2022). Additionally, aging-associated inflammation, or “inflammaging,” is a state of chronic low-grade inflammation caused by increased levels of pro-inflammatory cytokines and diminished ability to decrease inflammation. This inability to regulate the inflammatory immune response contributes to intestinal barrier dysfunction, leading to microbial imbalances and systemic inflammation (Weersma et al., 2020). The reduction in microbial diversity and SCFA production, along with an increase in pathobionts and a weakening of mucosal defenses contribute to an increase in susceptibility to gut and systemic diseases in the elderly. Addressing these factors through targeted intervention, such as dietary modifications, probiotics, and microbiota-preserving treatment, could aid in the support of gut and immune health in the elderly.

### Drug-induced alterations in gut microbiota and mucosal immunity

Drugs may contribute to or exacerbate age-related changes in gut microbiota and may impact mucosal immunity. Proton pump inhibitors, metformin, anti-inflammatory agents such as NSAIDS and steroids, and antibacterials play a significant role in disruption of gut mucosa. Additionally, 24% of marketed drugs inhibit at least one common intestinal microbiome bacterial strain, including nonantibacterial agents such as statins, angiotensin converting enzyme (ACE) inhibitors, atypical antipsychotics, and cholinesterase inhibitors. Polypharmacy, the use of multiple drugs, is also common in elderly patients with an average daily use of more than seven drugs by nursing home patients (Haran, et al., 2021). A summary of the classes, examples of agents, common indications for use, and noted microbiota association is found in Table 2.

**TABLE 2:** Drug Classes Associated with Alterations of Microbiota and/or Mucosal Immunity Proton pump inhibitors (PPIs) (Hodgkinson, et al., 2023; Trifan, et al., 2017), biguanide (Shin et al., 2014; Wu et al., 2017), NSAIDs (Gebril et al., 2020; Takeuchi, 2014; Wang et al., 2021; Maseda and Ricciotti, 2020), Corticosteroids (Tena-Garitaonaindia et al., 2022; Roca-Saavedra et al., 2022; Sáez-Lara et al., 2016; Meduri et al., 2025; Strasser et al., 2021), antibacterials (He et al., 2023; Panda et al., 2014; Bhalodi et al., 2019), statins (Dias et al., 2020), and antihypertensives (Yang et al., 2023).

Drug class	Examples (generic name)	Common indications	Microbiota association
Proton pump inhibitors	Omeprazole Pantoprazole Lansoprazole	Gastroesophageal reflux disease (GERD)	Increase in pathogenic bacteria and reduced production of SCFAs
Biguanide	Metformin	Type II diabetes mellitus	Increase in beneficial bacteria and modulation of gut permeability
NSAIDs	Ibuprofen Naproxen Indomethacin	Arthritis Headache Gout	Reduce mucus production, increase gut permeability, modulation of microbiota composition
Corticosteroids	Prednisone Dexamethasone Budesonide Methylprednisolone	Inflammatory conditions (e.g., infection, multiple sclerosis)	Increase in pathogenic bacteria, reduce mucus production, increase gut permeability
Antibacterials	Doxycycline Azithromycin Amoxicillin Clindamycin Meropenem	Bacterial infections	Variable based on spectrum, greatest risk of opportunistic pathogen overgrowth with broad spectrum agents
Statins	Atorvastatin Simvastatin Rosuvastatin	Dyslipidemia	Unknown, potentially shift microbiota composition toward beneficial bacteria
Antihypertensives	Lisinopril Losartan Amlodipine	Hypertension Heart failure	Unknown, may affect or be affected by hypertension or some drugs used to treat hypertension

### Proton pump inhibitors (PPIs)

These drugs decrease stomach acid production by irreversibly binding to H^+^/K^+^-ATPase (proton pump) in gastric parietal cells, preventing the ultimate step of gastric acid secretion. This inhibition leads to a significant and sustained increase in gastric pH, which disrupts the microbiota balance. This higher pH facilitates the overgrowth of microbes such as *Streptococcus* and *Rothia* in the lower gastrointestinal tract and pathogenic bacteria like *Clostridium difficile* (Imhann et al., 2016; Trifan, et al., 2017). Additionally, this environment reduces the production of short-chain fatty acids (SCFAs), which are essential for gut barrier integrity (Trifan, et al., 2017). Certain SCFAs, such as butyrate, help maintain tight junctions between epithelial cells by supporting epithelial cell energy metabolism. The depletion, however, of SCFAs lead to increased gut permeability (“leaky gut”) and heightens the risks of *Clostridium difficile,* leading to severe infections and diarrhea (Hodgkinson, et al., 2023; Trifan, et al., 2017).

### Metformin

Metformin is another agent that may disrupt the gut microbiota through direct epithelial and indirect microbial pathways. While it is used therapeutically to reduce hepatic glucose production by activating AMP-activated protein kinase (AMPK), a key regulator of energy metabolism, it also affects the gut microbiota by altering microbial composition and increasing mucus production. Metformin increases the abundance of *Akkermansia muciniphila*, a beneficial bacterium that strengthens the gut lining, by stimulating mucin secretion by goblets cells and enhancing mucin genes such as MUC2 through AMPK activation. This leads to a thicker and more diverse mucus layer that favors the increase of *Akkermansia muciniphila.* (Shin et al., 2014; Wu et al., 2017). *Akkermansia muciniphila* enhances the production of SCFAs, primarily butyrate and propionate. These SCFAs will activate G-protein coupled receptors GPR41 and GPR43 on intestinal and immune cells, which promotes anti-inflammatory cytokine (IL-10) release, reducing gut inflammation, and strengthening intestinal barrier function (Wu et al., 2017). Butyrate specifically inhibits histone deacetylases (HDACs), which causes an increase in histone acetylation leading to an increase in anti-inflammatory gene transcription. Additionally, metformin alters bile acid metabolism by modulating reabsorption through farnesoid X receptor (FXR) signaling in the ileum, promoting the expansion of beneficial taxa including *Blautia* and *Bifidobacterium* (Wu et al., 2017). This improved gut microbiota helps with regulating intestinal permeability, potentially reducing the risk of metabolic disorders such as type 2 diabetes and obesity (Petakh, et al., 2023).

### Non-steroidal anti-inflammatory drugs (NSAIDs)

This class of drugs, which includes both prescription and over-the-counter (OTC) agents, inhibits either or both cyclooxygenase-1 and -2 (COX-1,2) enzymes with variable selectivity, thereby blocking the conversion of arachidonic acid into prostaglandins. COX-1 is constitutively active and generates prostaglandins that maintain gastrointestinal mucosal integrity (by stimulating mucus/bicarbonate secretion and mucosal blood flow), whereas COX-2 is inducible and produces prostanoids during inflammation. By inhibiting both isozymes, these drugs reduce prostaglandin synthesis, particularly prostaglandin E_2_ and prostacyclin, which are gastroprotective (Takeuchi, 2014). In particular, COX-1 inhibition decreases gastrointestinal mucosal integrity and makes the surrounding epithelium more susceptible to injury. Reduced prostaglandin levels lead to decreased mucus production, diminished mucosal perfusion, and impaired maintenance of tight junctions, collectively resulting in reduced mucosal protection. Clinical and experimental evidence over the past decade supports this. For example, capsule endoscopy studies in chronic NSAID users show a high prevalence of subclinical small bowel injury: in one study, 71% of patients on >3 months of NSAIDs had small-intestinal mucosal damage, compared to only ∼10% of non-users (Wang et al., 2021). Intestinal tight junctions may be disrupted, demonstrated by elevated permeability on lactulose and mannitol tests and increased translocation of luminal contents. Rodents treated with the NSAID indomethacin exhibited “leaky” gut barriers, increasing intestinal permeability and inducing enteritis (Gebril et al., 2020). In addition to the effects of NSAIDs on the gut barrier, both human and animal studies link NSAIDs to gut dysbiosis due to decreased mucus production and antimicrobial properties of some NSAIDs. NSAID exposure tends to shift towards an increased relative abundance of Gram-negative bacteria (Maseda and Ricciotti, 2020). Clinical microbiome profiling has shown that NSAID users have an increased abundance of *Bacteroides*, Enterobacteriaceae, and other Gram-negative bacteria, alongside a decrease in beneficial genera like *Lactobacillus* and Bifidobacterium. Use of aspirin may be associated with increased relative abundance of Prevotella and *Bacteroides* in humans (Rogers and Aronoff, 2016).

### Corticosteroids

Corticosteroids, primarily prescribed for their anti-inflammatory effects, impact the gut microbiome by binding to the intracellular glucocorticoid receptor (GR), which displaces into the nucleus and modifies gene transcription, leading to a decreased production of pro-inflammatory cytokines such as TNF-α, IL-1, and IL-6. This suppression leads to a reduction in secretory immunoglobulin A (sIgA), an essential defense mechanism in mucosal immunity. SIgA aids in the neutralization of pathogens and maintenance of microbial homeostasis (Strasser et al., 2021; Roca-Saavedra et al., 2022). Although these effects inhibit inflammation systemically and locally in the gut, they can also impair mucosal immune surveillance and intestinal barrier integrity (Sáez-Lara et al., 2016). Glucocorticoids such as dexamethasone and prednisone have been found to impact gut microbial diversity by reducing the microbial content and increasing the abundance of opportunistic pathogens including Proteobacteria and Enterobacteriaceae, while reducing protective bacteria like *Lactobacillus* and Bifidobacterium (Meduri et al., 2025). Furthermore, corticosteroids inhibit goblet cell mucin secretion and inhibit epithelial repair mechanisms, resulting in a thinner mucus layer and increased intestinal permeability (“leaky gut”) (Tena-Garitaonaindia et al., 2022). This compromised barrier function may also exacerbate age-related gut barrier impairment. In animal models, prolonged corticosteroid exposure is associated with increased susceptibility to infections and colitis, potentially through pathways involving gut dysbiosis and weakened epithelial defenses (Meduri et al., 2025). While corticosteroids are essential in managing inflammation, their influence on the gut ecosystem must be carefully assessed, particularly in vulnerable populations such as older adults. Bacteria also affect activity of both endogenous and exogenous corticosteroids, and variable bacterial expression of genes such as *DesAB*, which produces a desmolase enzyme that oxidizes the tertiary alcohol of cortisol, contributes to bidirectional effects (effects of steroids on bacteria and effects of bacteria on steroid activity) (Zhang et al., 2024). Zhang et al. also detected significant differences in microbiota composition in Cushing’s Syndrome patients, a condition of excessive cortisol secretion, including decreased Bacteroidetes and increased Firmicutes, Actinobacteria, and Pseudomonadota.

### Antibacterials

Antibacterial antibiotics target fundamental bacterial processes such as cell wall or DNA synthesis. They are not inherently selective for pathogenic bacteria, and therefore indiscriminately deplete both pathogens and commensals (He et al., 2023). Gram-positive Firmicutes are vulnerable to, for example, certain beta-lactams, protein synthesis inhibitors, and glycopeptides, which effectively eradicate many Firmicutes (including beneficial *Lactobacillus* and *Clostridium* spp.) (Panda et al., 2014). Multiple antibacterials also cover Gram negative bacteria, such fluoroquinolones and aminoglycosides, and have activity against anaerobic Bacteroidetes such as *Bacteroides* and *Prevotella*. Antibacterial-driven dysbiosis is characterized by loss of microbial diversity and altered community structure (Zhang et al., 2018). Use of broad-spectrum antibacterials, or antibacterials that have activity against many Gram positive and Gram negative bacteria, can cause ∼25% reductions in gut bacterial diversity within days, often increasing the Bacteroidetes-to-Firmicutes ratio, as sensitive Firmicutes are depleted (Panda et al., 2014). The loss of competition and decreased colonization resistance in the microbiome affords the possibility of proliferation or less helpful or potentially harmful bacteria (Armstrong et al., 2025). Both clinical and animal studies indicate that microbiota recovery after broad-spectrum antibiotic exposure is sometimes incomplete (Bhalodi et al., 2019). Use of broad spectrum antibacterials is specifically associated with *C. difficile* infection. These findings show that broad spectrum antibiotics, while clinically necessary in some situations, may induce profound and long-lasting shifts in gut microbiota composition and function and should therefore be used cautiously (Tena-Garitaonaindia et al., 2022; Lee et al., 2023).

### Cardiovascular drugs

**FIGURE 2:**
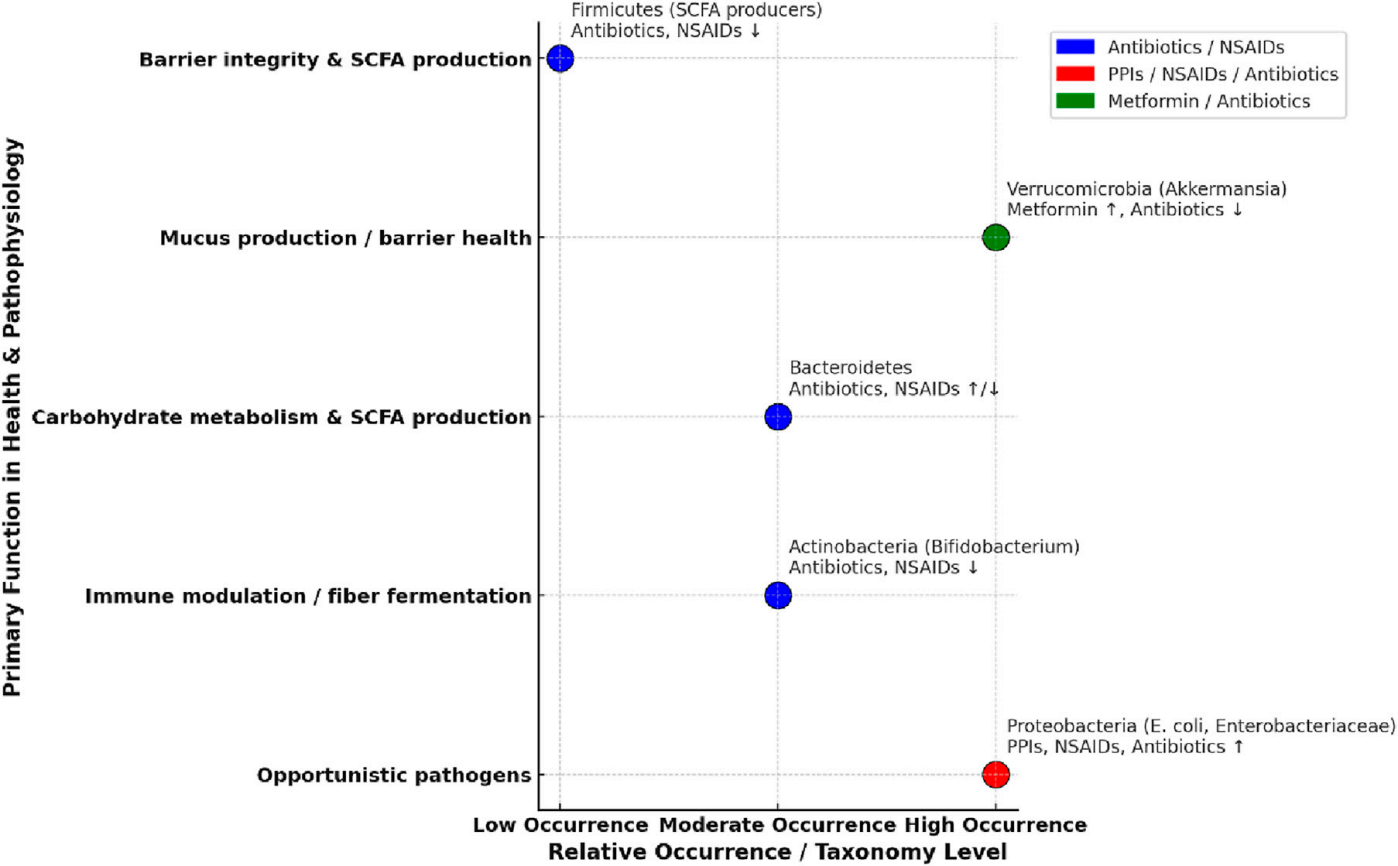
Age-related changes, gut health, and impact of drugs on gut microbiota. The horizontal axis depicts the general change in relative occurrence with aging, while the vertical axis lists major functions in gut health. (Weersma et al., 2020; Rogers and Aronoff, 2016; Wang et al., 2021; Bhalodi et al., 2019).

Medications for conditions such as dyslipidemia and hypertension may be associated with gut microbiota in either or both directions (i.e., the medication affects the microbiota or the microbiota affects the activity of the medication). Statins modify the composition and diversity of the gut microbiota, and their lipid-lowering effects may in turn be affected by gut bacteria. While not fully understood, potential mechanisms of bacteria-associated hypolipidemic activity may include drug metabolism, modulation of protein expression of enzymes associated with bile acid synthesis, or modification of drug transport. The mechanisms of effects of statins on gut bacteria are also not well understood and may involve an increase proportion of anti-inflammatory bacteria with a corresponding decrease in pro-inflammatory bacteria (Dias et al., 2020). Antihypertensive medications such as amlodipine and ACE inhibitors, as well as hypertension itself, may be associated with or affected by gut microbiota, and further study is needed to better understand the relationships between the condition, the drugs, and the microbiota (Yang et al., 2023). Relative change in abundance, role in gut health, and drug-bacteria interactions are depicted in Figure 2.

## Discussion

The dynamic relationship of gut microbiota, mucosal immunity, aging, and pharmaceutics interventions has a significant impact on overall physiological functions and disease susceptibility. Aging is associated with changes in the gut microbiome including decreased microbial diversity, reduced short-chain fatty acid (SCFA) production, and elevated pathobiont proportions (Zheng et al., 2022). These changes are associated with impaired mucosal immunity, increased intestinal permeability, and heightened systemic inflammation in the host, which can exacerbate age-related disorders (Clark and Walker, 2018).

Medications such as proton pump inhibitors (PPIs), metformin, nonsteroidal anti-inflammatory drugs (NSAIDs), corticosteroids, and antibacterials also influence gut microbiota function. PPIs may alter microbial colonies and induce overgrowth of pathogenic bacteria, which compromises the mucosal defenses and in severe cases, the resulting infections may cause ulcers (Kavitt et al., 2019). Metformin, through its metabolic benefits, causes *Akkermansia muciniphila* to increase in relative abundance, which is associated with improved gut barrier composition. NSAIDs, because of their strong anti-inflammatory properties, disturb gut homeostasis by increasing intestinal permeability, reducing prostaglandin synthesis, and inducing dysbiosis in the host. Corticosteroids, through their immunosuppressive mechanisms, reduce microbial diversity and secretory immunoglobulin A (sIgA) levels, impairing mucosal immunity and enhancing the host’s susceptibility to infections. Antibacterials are a major disruptor of the gut microbiota, causing a decline in beneficial bacteria and an increased risk for opportunistic infections such as *Clostridium difficile*.

To address drug-induced dysbiosis, probiotics and prebiotics products may be helpful to restore microbial balance, enhance SCFA production, and reinforce mucosal defenses (Sarita et al., 2025). Individualized gut microbiota profiling may enable safer medication usage by identifying patients that are at an increased risk for dysbiosis-related complications (Shukla et al., 2024). Additionally, development of microbiota-sparing medications and targeted therapies may help to enhance gut health outcomes in aging populations (Avis et al., 2021).

Future research should address the long-term effects of pharmacological agents on gut microbiota and mucosal immunity in aging populations, as well as identification of connections between microbiota, immune function, and the effects of medications. Integrating microbiome-conscious approaches into clinical practice could allow healthcare providers to optimize patient care (Avis et al., 2021).

## Conclusion

The connection between gut microbiota, mucosal immunity, aging, and pharmaceutical medications is critical to maintaining health and reducing the disease susceptibility of the aging population for maintenance of health and to decrease the risk of diseases such as CRC. Medications such as PPIs, NSAIDs, corticosteroids, metformin, and antibiotics alter the composition of the gut microbiota through complex and sometimes bidirectional mechanisms. Preservation of microbial diversity and mucosal barriers through the usage of probiotics, prebiotics, and microbiota-sparing drug therapies may amend such effects and warrant further investigation. Gut microbiota profiling and genomic analysis could individualize therapies to both improve microbial diversity and minimize the risk of dysbiosis complications for treatment of other conditions. Further research is needed to explore strategies that maintain or improve microbial balance and immune competency to improve treatment outcomes and the long-term health of the aging population.
